# The Effectiveness of Interventions to Reduce Sedentary Time in Different Target Groups and Settings in Germany: Systematic Review, Meta-Analysis and Recommendations on Interventions

**DOI:** 10.3390/ijerph191610178

**Published:** 2022-08-17

**Authors:** Nida Mugler, Hansjörg Baurecht, Kevin Lam, Michael Leitzmann, Carmen Jochem

**Affiliations:** Department of Epidemiology and Preventive Medicine, University of Regensburg, 93053 Regensburg, Germany

**Keywords:** sedentary behavior, interventions, Germany, systematic review, meta-analysis

## Abstract

Background: Sedentary behavior is an important risk factor for several chronic diseases and is associated with an increased risk of mortality. We assessed the effectiveness of interventions to reduce sedentary time in Germany and provide recommendations on interventions to reduce sedentary time in children and adults. Methods: We comprehensively searched PubMed, Web of Science and the German Clinical Trials Register up to April 2022 for intervention studies targeting sedentary behavior in Germany. We performed a systematic review and qualitative synthesis of the interventions and a meta-analysis in children. Results: We included 15 studies comprising data from 4588 participants. The results of included primary studies in adults and children showed inconsistent evidence regarding change in sedentary time, with a majority of studies reporting non-significant intervention effects. The meta-analysis in children showed an increase in sedentary time for children in the control and intervention groups. Conclusion: We found inconsistent evidence regarding the effectiveness of interventions to reduce time spent sedentary and our meta-analysis showed an increase in sedentary time in children. For children, we recommend physical and social environment interventions with an active involvement of families. For adults, we recommend physical environment interventions, such as height-adjustable desks at work.

## 1. Introduction

Sedentary behavior is defined as “any waking behavior characterized by an energy expenditure ≤1.5 metabolic equivalents (METs), while in a sitting, reclining or lying posture” [1]. Sedentary behavior is an emerging risk factor for several chronic diseases, including cardiovascular disease [2,3], type 2 diabetes [3,4,5] and various tumor entities [6,7] which contribute to the global burden of disease [8]. It is also associated with an increased risk of overweight and obesity among adults [4], reduced (health-related) quality of life [9,10] and an increased risk of depression [11,12]. Furthermore, sedentary behavior is associated with an increased risk of all-cause mortality [13,14] and it causes substantial health care costs [15]. Altogether, sedentary behavior is associated with various aspects of health and well-being and should therefore be considered in prevention measures.

Globally, the prevalence of time spent sedentary has increased over the past two decades [16,17], a trend that is also present in Germany [18,19]. In 2012, the median self-reported sitting time was 300 min per day for men and 240 min for women in Germany [18] and it increased to a median sitting time of 480 min for men and 425 min for women in 2018 [19]. Internationally, the estimated objectively measured sedentary time is 9.4 h per day in older adults (≥60 y) [20] and 8.2 h per day in adults [21]. The averaged estimate of objectively and subjectively measured sedentary time is 9.3 h per day in adolescents and 4.4 h per day in children [21].

Different intervention approaches can be implemented to reduce sedentary time. Common strategies are the introduction of activity permissive workstations (i.e., interventions targeting the physical environment) or interventions targeting personal behavior (for example, through the transfer of knowledge or the use of pedometers) [22]. Several reviews and meta-analyses have summarized international findings of interventions aimed to reduce sedentary time in specific target groups or in specific settings [22,23,24,25].

Although working and living conditions in Germany are comparable to those in other Western countries in many aspects [26,27], there are some differences which may conceivably influence sedentary behavior interventions. An above-average proportion of children under three years are cared for solely by their parents in Germany [27] and also school-aged children up to 12 years spend below-average time in formal daycare [27]. Therefore, it may be necessary to focus more on interventions targeting the family as the social environment in this age group and institution-based interventions may be less effective. In recent years, the average annual hours worked in Germany were below average (compared to other developed countries) [28] which might lead to reduced effectiveness of work-based interventions. Due to considerable international variation in time spent sedentary [16,29] and the outlined differences in working and living conditions, we identified a need to assess the effectiveness of sedentary behavior interventions in Germany specifically.

The current systematic review and meta-analysis aimed at investigating the effectiveness of interventions to reduce sedentary time in different target groups and different settings in Germany. An additional goal was to provide specific recommendations on the effectiveness of different types of interventions for different target groups and different settings in Germany in order to contribute to future guideline development processes. The recommendation of effective types of interventions that may reduce sedentary time is especially important for public health to inform future guidelines that aim to decrease sedentary time from a public health perspective.

## 2. Materials and Methods

This present study was performed following the Preferred Reporting Items for Systematic Reviews and Meta-Analyses (PRISMA) 2020 statement [30]. The PRISMA Checklist can be accessed in Supplementary Figures S1 and S2.

### 2.1. Inclusion and Exclusion Criteria

To be included in the systematic review, studies had to fulfill the following inclusion criteria:

- Study design: included studies needed to be controlled intervention studies (e.g., randomized/non-randomized controlled studies, cross-over randomized/non-randomized studies, cluster randomized/non-randomized controlled studies, controlled before and after studies).
- Population: participants of all ages, with and without chronic diseases were included.
- Intervention: all types of interventions (interventions targeting the physical or social environment, personal behavior and multicomponent interventions) aiming at reducing sedentary time were deemed eligible, irrespective of whether sedentary time was the primary or secondary outcome.
- Control: any control condition was considered eligible; we included controls without intervention, waitlist controls and controls with an alternative intervention.
- Outcome: we included sedentary behavior across different domains of sitting or sedentary time.
- Setting: at least part of the intervention needed to have been implemented in Germany and results had to have been reported separately for Germany.

We included peer- and non-peer-reviewed studies (irrespective of language) and with any country of origin as long as the study reported data obtained in Germany.

We excluded observational studies, studies without a control condition and interventions implemented in countries other than Germany. Sedentary behavior domains such as screen time, media consumption and individual sedentary activities (e.g., doing homework) were excluded. We excluded pharmacological interventions, animal studies and cross-sectional studies. No date or language restrictions were applied.

### 2.2. Search Strategy

We systematically searched the databases PubMed/Medline, Web of Science and the German Clinical Trials Register (DRKS) from inception through April 2022. Additionally, we hand-searched reference lists and intervention programs of included studies. We contacted authors of studies where interventions were implemented in multiple countries including Germany but data were not reported separately for Germany. We developed search terms comprising sedentary behavior and related interventions (Supplementary Tables S1–S3).

### 2.3. Study Selection Process and Data Extraction

Titles and abstracts of potentially eligible articles were screened by one author (NM) and in unclear cases a decision was made after consultation with a second author (CJ). Subsequently, two authors (NM, CJ) assessed the remaining full-text articles and independently decided about their inclusion in the review. When disagreements arose, a third author (ML) was contacted. The authors were not blinded to the title and authors of the potentially eligible articles.

Two authors (NM, CJ) agreed on an Excel extraction table that covered all relevant items. One author (NM) extracted the data of included studies and a second author (CJ) independently verified the data. Potential disagreements were resolved by a third author (ML). The following items were extracted: title, first author’s name, year of publication, name of intervention program, study design, study population (babies, toddlers, preschoolers, school children, teenagers, adults, older adults, office workers, people with chronic diseases, obese and overweight people), sex, age, number of participants, country, region, ethnicity, intervention focus, domain of sedentary behavior (daycare, school, training facility, work, office, domestic environment, transportation, leisure time, total), timing and length of sedentary behavior (baseline, latest follow-up), change in sedentary behavior between measurements (baseline, latest follow-up), difference in sedentary behavior change between groups (baseline, latest follow-up), description of intervention and control condition, intervention category (personal behavior, physical environment, social environment, multicomponent), intervention length, points of measurement, outcome measurement (type of instrument, quantitative, qualitative assessment), adjustment for potential confounders, funding source, country of origin (location of the institution of the corresponding author), peer-review status, language and protocol or registration number.

We categorized interventions into those targeting the social environment (e.g., targeting parents of children, walking meetings), interventions targeting the physical environment (e.g., height-adjustable desks, movement-friendly playgrounds), interventions targeting personal behavior (e.g., coaching, fitness tracker) and multicomponent interventions targeting at least two of the aforementioned areas.

### 2.4. Data Synthesis

We performed a systematic review and qualitative synthesis of the interventions for adults and children. We grouped studies according to their target population to allow comparison between interventions and graphically presented results of included studies. As the identified studies in adults systematically differed regarding study population, outcome and type of intervention, we refrained from combining the results in a meta-analysis.

To quantify total sedentary or sitting time in children in the intervention and control groups before and after the intervention, we conducted a random effects model (REM) meta-analysis because heterogeneity was expected due to different settings, study designs and interventions. We meta-analyzed primary studies reporting pre- and post-intervention sedentary or sitting time in minutes per day (quantitatively or qualitatively measured). Studies that reported units which could be converted into minutes per day were also included in our meta-analysis. We included primary studies if the standard error (SE) was given or could be calculated from the standard deviation, the 95% confidence interval or the 25th/75th percentile, assuming a normal distribution. We determined study heterogeneity using the I2-statistics [31]. We performed a subgroup analysis according to the different intervention categories (multicomponent and physical environment) in children.

All statistical analyses were conducted using the software R (version 4.0.5) (Vienna, Austria) [32], using the packages “metafor” (version 2.4-0) (Maastricht, Netherlands) and “meta” (version 4.16-2) (Freiburg, Germany) to conduct the meta-analyses. The code is available from the authors on request.

### 2.5. Risk of Bias Assessment

To determine validity of included studies, we assessed the risk of bias in included studies. Accordingly, we used the revised Cochrane risk-of-bias tool for randomized trials (RoB 2) [33] to assess risk of bias. We evaluated the following domains for each study: randomization process, deviations from the intended interventions, missing outcome data, measurement of the outcome, selection of the reported result. For cluster-randomized trials, we used the RoB 2 CRT tool and additionally judged the domain timing of identification or recruitment. For randomized cross-over trials, we used the version adopted for this purpose and additionally judged the domain period and carryover effects. Each domain was judged as high risk, low risk or some concerns and thus an overall risk of bias judgement was built for each study.

## 3. Results

### 3.1. Study Selection

**Figure 1:**
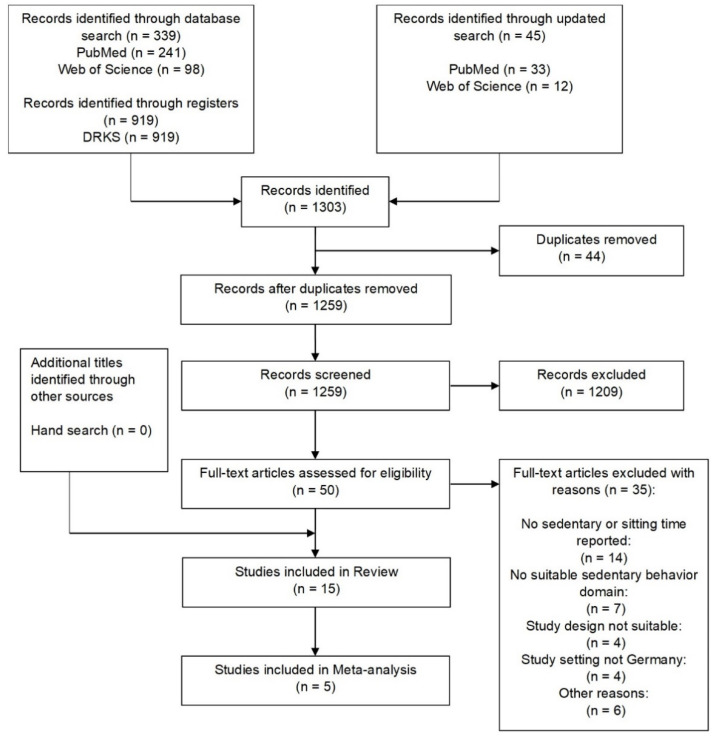
PRISMA flow diagram depicting the process of study selection. n = number of articles. DRKS = German Clinical Trials Register.

Our systematic database search retrieved 384 studies. Of those, we retrieved 274 studies through PubMed/Medline and 110 studies through Web of Science. We identified 919 additional studies through DRKS. Figure 1 depicts the literature search according to the PRISMA guidelines. After removal of duplicates, we screened 1259 studies for potential eligibility. Of those, we excluded 1209 studies because they did not report a suitable sedentary behavior domain, had an inappropriate study design or were not implemented in Germany. We assessed 50 full-text articles for eligibility, of which 35 were excluded. The reasons for exclusion of those full-text articles were that sitting time or sedentary time was not reported [34,35,36,37,38,39,40,41,42,43,44,45,46,47] or an unsuitable domain of sedentary behavior was reported [48,49,50,51,52,53,54], the study design was not suitable [55,56,57,58], the study setting was not Germany [59,60,61,62] or various other reasons [63,64,65,66,67,68]. A complete list of reasons for exclusion can be found in Supplementary Table S4. No additional eligible articles were identified through manual search. The fifteen remaining studies were included in our qualitative synthesis and five studies were included in our meta-analysis.

### 3.2. Risk of Bias Assessment

Ten studies [69,70,71,72,73,74,75,76,77,78] referred to published protocols [79,80,81,82,83,84] or trial registry records (trial IDs reported in Table 1) which were consulted in these cases. Nine studies [69,70,73,76,77,78,85,86,87] were judged to be of high risk of bias and four studies [71,72,74,75] to have some concerns. No studies were judged to be of low risk of bias. Detailed judgement of the individual domains in each study can be found in Supplementary Table S5.

### 3.3. Study Characteristics

The main characteristics of the 15 included studies are summarized in Table 1. In two instances [88,89], the authors referred to an additional paper containing more detailed information on the respective study [90,91]. Of the 15 included studies, 14 studies [69,70,71,72,73,74,75,76,77,78,85,87,88,89] were published in peer-reviewed journals, 14 were written in English [69,70,71,72,73,74,75,76,77,78,85,87,88,89] and one [86] in German. The country of origin was Germany for 13 studies [69,70,71,73,74,75,76,77,78,85,86,87,88] and Belgium [89] and the UK [72] for one study each.

Two studies [72,89] were implemented in several European countries. For those studies, we only included data of participants who received their intervention in Germany. The remaining studies were exclusively implemented in Germany.

**Table 1:** Characteristics of included studies.

Authors, Year, Funding Source, Trial Registry	Study Design, Health Promotion Program	Population	Intervention Duration, Follow-Up	Intervention Setting, Type of Intervention, Intervention Focus	Intervention Description	Control Condition	Assessment of Outcome, SB Outcomes
**Children**							
Brandes et al., 2020 [88] Steenbock et al., 2017 [90] “ German health insurance AOK DRKS00011065	Cluster non-RCT JolinchenKids (JOKITA; fit and healthy in daycare)	Preschoolers, n = 144, age range: 3–6 y IG: n = 67, mean age: 4.1 y, gender: 46.8% male, 53.2% female CG: n = 77, mean age: 4.3 y, gender: 49.4% male, 50.6% female	1 year 1 year	Daycare based Multicomponent intervention PA	Five modules: three focused on children (one on PA, nutrition and mental well-being each), one on parents, one on DF staff PA module: instructions for PA games, aim to create movement-friendly areas in DF, additionally: parents received newsletter once yearly and participated in joint activities twice yearly	Wait list	Quantitative (GENEActiv device) Domain SB: total sedentary behavior (min/day)
De Bock et al., 2013 [75] Grant from Baden-Württemberg Stiftung * NCT00987532	Cluster RCT Ene mene fit	Preschoolers, n = 528, age range: 4–6 y mean age: 5.05 y, gender: 52% boys, 48% girls IG: n = 248 CG: n = 280	IG: 9 months CG: 6 months 6 months, 12 months	Daycare community based Multicomponent intervention PA	Non-participatory PA program: one-hour gym class (twice weekly over 6 months), one meeting of parents and gym class trainer Additionally: participatory intervention, incorporating parents, teachers and other preschool community members, website (www.ene-mene-fit.de (accessed on 2 July 2021)), introductory video, book with project ideas, gym trainers provided support to parents and preschool communities in implementing own interventions	Only non-participatory PA program	Quantitatve (Actiheart) Domain SB: total sedentary behavior (min/day)
Verbestel et al., 2015 [89] ** Ahrens et al., 2011 [91] “ n/a	Cluster non-RCT Identification and prevention of Dietary- and lifestyle-induced health EFfects In Children and infantS (IDEFICS)	Toddlers, preschoolers, school children, n = 1097, age range: 2–9.9 y	2 years 2 years	Community, school and family based Multicomponent intervention SB/PA	Ten intervention modules: - Modules one to three: community level, comprised community platform, media campaign, public relation strategy, policy interventions - Modules four to nine: school level, comprised school working groups, education of the children (eight “healthy weeks” dedicated to specific topic during the school year), environmental, curricular, school policy changes to foster PA, water, fruit and vegetable consumption - Module ten: family level, aimed at education of parents	No intervention	Quantitative (ActiGraphTM GT1 M and ActiTrainerTM) Domain SB: total sedentary time (% of time)
Kobel et al., 2020 [78] Funded by Baden-Württemberg Foundation DRKS00000494	Cluster RCT Join the Healthy Boat	School children, n = 154, age range: 5–8 y IG: n = 102, mean age: 7 y, gender: 48% male, 52% female CG: n = 52, mean age: 7 y, gender: 42.3% male, 57.7% female	1 year 1 year	School based Multicomponent intervention PA/SB	Primary school teachers trained to conduct change towards movement-friendly school environment and to promote healthy and active lifestyle in lessons, implemented short exercises twice daily to break up SB, held teaching units once weekly (containing 20 lessons dealing with health-related topics, 13 focused on PA and SB). To encourage parental involvement: six family homework assignments, two parents´ nights, additionally: parents received five letters (three were dealing with PA and SB)	No intervention, continued usual school curriculum	Quantitative (Actiheart) Domain SB: total sedentary time total week (min/day) Sedentary time weekdays (min/day) Sedentary time weekend (min/day)
Sprengeler et al., 2020 [87] City of Ludwigsburg: height-adjustable standing desks, publication of article funded *	Cross-over RCT n/a	School children, n = 37, Age range: 8–10 y mean age: 8.4 y, gender: 38.5% boys, 61.5% girls G1: n = 19 G2: n = 18	2 weeks (each for G1, G2) 2 weeks, 9.43 weeks	School based Physical environment SB	First G1 received height-adjustable standing desks and G2 served as control. After washout period, G2 used height-adjustable standing desks and G1 served as control	Respective group serving as control used traditional working desks	Quantitative (activPAL inclinometer) Domain SB: total, school, leisure time Sitting time (min/day) Sitting time (% of total time during lessons, % of total time during breaks, % of total time during leisure time)
Suchert et al., 2015 [76] German Cancer Aid: Primary Prevention of Cancer ISRCTN49482118	Cluster RCT läuft	Teenagers, n = 1162, age range: 12–17 y IG: n = 702, mean age: 13.7 y, gender: 52.7% male, 47.3% female CG: n = 460, mean age: 13.79 y, gender: 50.9% male, 49.1% female	12 weeks 12 weeks	School based Multicomponent intervention PA	Pedometers, could record and compare their steps on “läuft” homepage Classes participated in two competitions, attended educational lessons, schools and parents received information material	Regular education	Qualitative (survey) Domain SB: total sedentary behavior (h/day)
**Adults**							
Voigt et al., 2018 [70] Federal Ministry of Education and Research NCT02990039	RCT n/a	Adults, n = 138, age range: 40–65 y, mean age: 54.5, gender: 35.9% male, 64.1% female IG1: n = 69 IG2: n = 69	4 months (after split of IG1 and IG2) 1 month, 6 months, 12 months	Home based Personal behavior PA/SB	During first month, IG1 and IG2 were not split up, had an assessment only After separation: IG1 received letters at month 1, 3, 4 (letters were tailored via self-report assessing personal PA and SB behavior)	After separation, participants in IG2 received no letters (only assessed their PA and SB behavior via self-report)	Qualitative (IPAQ) Domain SB: total overall sedentary time (hours/week and sqrt min/week)
Livingstone et al., 2020 [72] ** Work supported by European Commission under the Food, Agriculture, Fisheries and Biotechnology Theme * NCT01530139	RCT Food4Me	Adults IG: n = 63	6 months 6 months	Web based Personal behavior PA	Participants randomized to intervention groups L1–L3, all participants received personalized dietary and PA advice but based on different sources (tailored feedback reports at baseline, month three, month six) L1: advised based on current PA and diet L2: additionally advised based on phenotypic data L3: additionally advice based on phenotypic and genotypic data	General (non-personalized) dietary and PA advice	Quantitative (triaxial accelerometer (Tracmor D)) Domain SB: total % of participants benefiting from the intervention (>5% reduction concerning sedentary time from baseline to month 6)
Ellegast et al., 2012 [85] German Social Accident Insurance (DGUV)	RCT n/a	Office workers, n = 25, gender: 76% male, 24% female IG: n = 13 CG: n = 12	12 weeks 12 weeks	Work based Multicomponent intervention PA/SB	Intervention at the workplace (office), intervention included sit–stand tables, pedometers, face-to-face motivation for, e.g., lunch walks, incentives for bicycle commuting/sports activities	No intervention	Qualitative (activity log), quantitative (CUELA Activity System) Domain SB: office sitting time (min/day) Tasks carried out sitting (%)
Pischke et al., 2022 [77] Federal Ministry of Education and Research (project 01EL1822A, 01EL1822F, 01EL1822l and 01EL1822C) DRKS00016073	Cross-over randomized trial PROMOTE II	Older adults, n = 160, age range: 60–82y IG1 (WEB): n = 59, mean age: 67.9 y, gender: 36% male, 64% female IG2 (WEB^+^): n = 22, mean age: 70.5 y, gender: 53% male, 47% female IG3 (PRINT): n = 79, mean age: 67.6 y, gender: 24% male, 76% female	9 months 3 months 9 months	Web and community based Personal behavior PA	WEB intervention group: online WHO PA recommendations, online brochures with instructions for exercises, online PA diary with weekly feedback, smartphone app with additional access to PA diary and exercises, additionally: 90 min weekly group session for 10 weeks After 10 weeks, participants had the option to cross over to another group, then group sessions continued once per month for 6 months WEB^+^ intervention group: same intervention and intervention materials online as participants in WEB group, additionally: PA tracker	PRINT group: same intervention and intervention materials as participants in WEB and WEB^+^ group but in printed form, did not receive PA tracker	Quantitative (GT3 x+) Domain SB: total sedentary time (min/day) Sedentary time in 30 min bouts (min/week)
Muellmann et al., 2019 [71] German Federal Ministry of Education and Research DRKS00010052	RCT PROMOTE study	Older adults, n = 405, age range: 62–79 y IG1: n = 146, mean age: 69.6 y, gender: 46.2% men, 53.8% women IG2: n = 119, mean age: 69.6 y, gender: 41.3% men, 58.7% women CG: n = 140, mean age: 69.8 y, gender: 42.6% men, 57.4% women	10 weeks, 12 weeks	Web and community based Personal behavior PA	IG1: Access to website, weekly group meetings in their community led by trained staff, additionally: instruction brochures tailored to their PA levels and gender, were instructed to exercise regularly Functions of the website: track PA, weekly feedback on predetermined PA goals, online rewards if goals were reached, option to contact other participants IG2: Same intervention as IG1, additionally: Fitbit Zip	Delayed intervention (access to web-based PA intervention, but did not receive Fitbit or group meetings)	Quantitative (ActiGraph GT3 X+) Domain SB: total sedentary time (min/day) Sedentary time in 30 min bouts (min/week)
Kleinke et al., 2021 [69] Federal Ministry of Education and Research (funding sign: 81Z7400174) DRKS00010410	RCT Motivation-Oriented intervention study for the elderly in Greifswald (MOVING-study)	Older adults, n = 166 IG: n = 85, mean age: 70.4 y, gender: 41.2% male, 58.8% female CG: n = 81, mean age: 71.2 y, gender: 42% male, 58% female	14 weeks 3 months 6 months	Home based Personal behavior PA/SB	Personalized feedback (based on accelerometer data) regarding their PA and SB via mail (after baseline and the 3-month follow-up examination), additionally: WHO PA recommendations	Did not receive any intervention, but received feedback concerning their accelerometer data after end of study	Quantitative (ActiGraph GT3 x-BT) Domain SB: total sedentary time (min/week)
Salchow et al., 2021 [73] Authors received no funding DRKS00009453	RCT Motivate Adolescent and Young Adults (MAYA)	Adults, people with chronic diseases (cancer survivors), n = 69, age range: 15–39 y IG: n = 36, mean age: 23.4 y, gender: 36.1% male, 63.9% female CG: n = 33, mean age: 25.3 y, gender: 48.5% male, 51.5% female	12 weeks 12 weeks 52 weeks	Home based Personal behavior PA	Sixty minutes of personally tailored PA counselling, containing a PA anamnesis and the development of a personalized PA plan, additionally: PA guidelines for cancer survivors and list with local opportunities for PA	Usual care (consisting of the PA guidelines for cancer survivors)	Qualitative (IPAQ) Domain SB: total sitting (hours/day)
Geidl et al., 2021 [74] German Pension Insurance * NCT02966561	RCT Stay Active after Rehabilitation (STAR)	Adults, people with chronic diseases (COPD), n = 327 IG: n = 167, mean age: 58.01 y, gender: 68.7% male, 31.3% female CG: n = 160, mean age: 58.03 y, gender: 69.4% male, 30.6% female	3 weeks 11 weeks 31 weeks	Stationary rehabilitation Personal behavior PA	Three-week stationary pulmonal rehabilitation, additionally, pedometer and two 45 min PA lessons	Same 3-week stationary rehabilitation as intervention group, instead of pedometer and PA lessons: repetition of PA information twice for 45 min	Quantitative (ActiGraph wGT3X) Domain SB: total sedentary time (min/day)
Wagner et al., 2019 [86] German Social Accident Insurance (DGUV) DRKS00010777	RCT n/a	Adults, people with chronic diseases (occupational respiratory diseases), n = 137, age range: 45–80 y, mean age: 69.1 y, gender: 95.9% male, 4.1% female IG: n = 67 CG: n = 70	IG: 7 weeks CG: 4 weeks 4.53 months 8.53 months 14.53 months	Stationary rehabilitation Personal behavior PA	Standardized stationary rehabilitation (similarly to CG) and additional behavior-orientated movement intervention (three weeks, contained three 45 min group sessions per week, tailored work folder with tasks)	Four-week stationary, standardized rehabilitation (included personally tailored interventions and activities)	Quantitative (Actigraph GT3 x) Domain SB: total sedentary behavior (min/day)

CG = control group, DF = daycare facility, G = group, h = hour, IG = intervention group, IPAQ = International Physical Activity Questionnaire, L = level, min = minute, n = number of participants analyzed in full intervention, PA = physical activity, RCT = randomized controlled trial, SB = sedentary behavior, y = year. * Details not reported at this point but can be looked up in the publication, “ contains additional information, referred to in the included study. ** pan-European studies, in these cases we only included data of participants who received their intervention in Germany.

The included studies comprised data from 4588 participants that were analyzed throughout the full course of the intervention. One study [72] did not report the exact number of participants included. One study focused on toddlers [89], three on preschoolers [75,88,89], three on school children [78,87,89], one on teenagers [76], two on adults [70,72], three on older adults [69,71,77], one on office workers [85] and three on people with chronic diseases [73,74,86].

The intervention duration was less than six months in nine studies [69,70,71,73,74,76,85,86,87], at least six months but less than twelve months in three cases [72,75,77] and it lasted twelve months or longer in three studies [78,88,89]. Five studies encompassed follow-up after the outcome assessment at the end of the intervention to assess long-term effects [70,73,74,75,86]. Six studies examined multicomponent interventions [75,76,78,85,88,89], eight studies focused on personal behavior interventions [69,70,71,72,73,74,77,86] and one targeted the physical environment [87]. No study examined the social environment. In fourteen studies, the principal sedentary behavior domain studied was total sedentary time [69,70,71,72,73,74,75,76,77,78,86,87,88,89]. One study each focused on sedentary time at school [87], in the office [85] and during leisure time [87]. Twelve studies [69,71,72,74,75,77,78,85,86,87,88,89] assessed sedentary time quantitatively (e.g., by wearing an accelerometer) and four studies [70,73,76,85] assessed sedentary time qualitatively (e.g., by questionnaire).

### 3.4. Description and Effectiveness of Interventions

**Figure 2:**
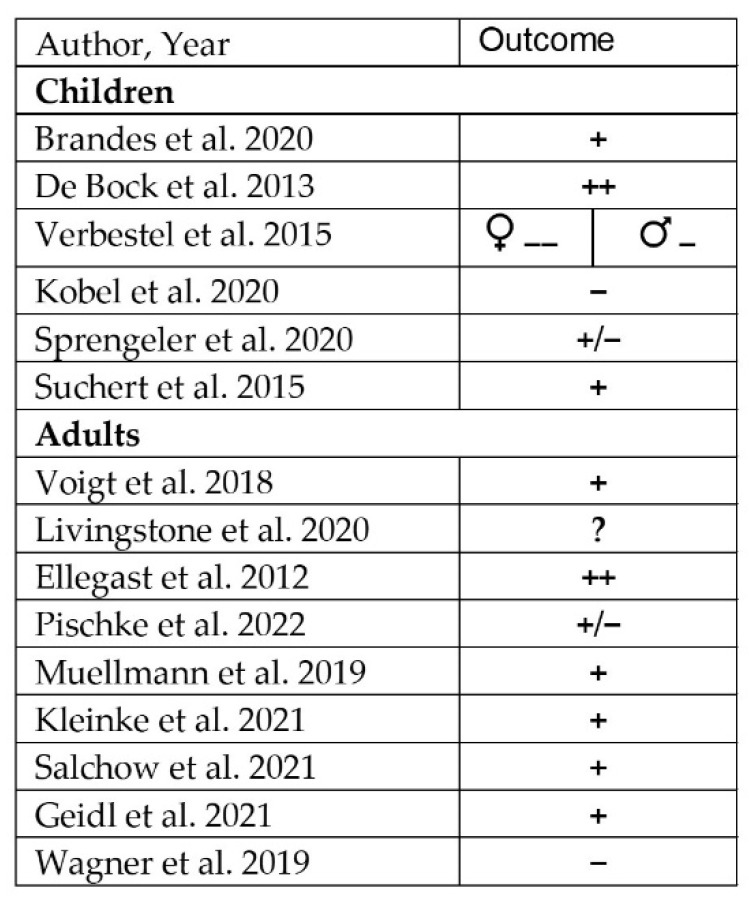
Summary of results of studies included in systematic review [69,70,71,72,73,74,75,76,77,78,85,86,87,88,89]. ++ significant reduction, −− significant increase, + tendency towards reduction in sedentary time, − tendency towards increase in sedentary time, ? significance cannot be assessed, +/− mixed results.

A graphical visualization of the results of the included studies is shown in Figure 2.

#### 3.4.1. Adults

Nine studies investigated the effectiveness of an intervention in adults. One study [85] showed a significant decrease in sedentary time for intervention group participants, one study showed a significant group effect [69], six studies did not show a significant change in time spent sedentary [70,71,73,74,77,86] and for one study [72] the statistical significance of the results could not be assessed because the corresponding *p*-value and confidence interval were not given.

Three studies assessed the effectiveness of interventions in adults with a mean age under 65 years. Two of them implemented a personal behavior intervention [70,72] and one implemented a multicomponent intervention [85].

At the 12-month follow-up of the first study [70] targeting adults aged less than 65 years, both groups showed reduced sedentary time compared to baseline, with participants in the group serving as control spending less time sedentary than intervention group participants. Nevertheless, no statistically significant difference in change in overall sedentary time between groups (*p* = 0.109) was shown. Results of the second study [72] showed that at six months, 42.9% of participants randomized to an intervention group benefited from the intervention (>5% reduction in sedentary time from baseline to month 6).

A third study [85] targeting office workers showed that at the 12-week follow-up, participants in the intervention group spent statistically significantly (*p* ≤ 0.001) less time sitting per day during work compared to controls.

Three studies enrolled older adults (mean age > 65 years) for a personal behavior intervention [69,71,77].

At the 9-month follow-up of the first study [77] targeting older adults, only participants in the second intervention group reduced their sedentary time compared to baseline while time spent sedentary increased in the two other groups (IG1: 630.2 min/day to 638.1 min/day, IG2: 637.7 min/day to 628.9 min/day, IG3: 639 min/day to 646.8 min/day). Nevertheless, changes in sedentary time did not differ significantly between groups (IG1: β = 10.41, 95% CI −4.49 to 25.31, IG2: β = −0.13, 95% CI −19.49 to 19.22) compared to the third intervention group (serving as control group). Results of the second study [71] targeting older adults showed that participants in all groups decreased their daily sedentary time from baseline to follow-up (IG1: 722.3 min/day to 693.8 min/day, IG2: 723.9 min/day to 697.7 min/day, CG: 705.6 min/day to 703.1 min/day). However, changes did not differ significantly between intervention groups 1 and 2 participants from baseline to follow-up compared to control group participants (IG1: β = 6.27, 95% CI −1.32 to 13.87, IG2: β = 0.32, 95% CI −7.67 to 8.30). Comparing time spent sedentary per day in adults in both intervention groups, results showed the benefit to the second intervention group but did not reach statistical significance (*p* = 0.24). At the 6-month follow-up of the third study [69] targeting older adults, participants in the intervention and control groups both showed reduced sedentary time compared to baseline (IG: 3482 min/week to 3354.8 min/week, CG: 3438.5 min/week to 3228.2 min/week), with a significant difference between groups (*p* = 0.022).

Another three studies implemented personal behavior interventions in adults with chronic diseases [73,74,86]. At the 12-month follow-up in the intervention among cancer survivors [73], results showed no significant difference (*p* = 0.148) between intervention and control group participants and both groups showed reduced sitting time from baseline to follow-up, although control group participants almost returned to baseline sitting levels (IG: 6.4 ± 3.0 h/day to 5.7 ± 2.3 h/day, CG: 6.6 ± 2.9 h/day to 6.4 ± 2.6 h/day). Another study [74] targeting people with COPD did not reveal significant differences (*p* = 0.988) between the intervention and control group at the 31-week follow-up, but participants in both groups spent less time sedentary compared to baseline (IG: 563.8 min/day to 551.6 min/day, CG: 559.1 min/day to 548.2 min/day). At follow-up 12 months after the end of the intervention among adults with occupational respiratory diseases [86], intervention group participants increased their sedentary time compared to baseline (499.4 min/day to 516.4 min/day) while control group participants reduced time spent sedentary (514.7 min/day to 500.6 min/day). Results did not differ significantly (*p* = 0.281).

#### 3.4.2. Children and Adolescents

Of the six studies targeting children aged 2 to 17 years, one study [75] showed a statistically significant reduction in time spent sedentary in intervention group participants. One study [89] reported statistically significant increased time spent sedentary in girls in the intervention group and one study [87] showed mixed results. Three studies did not report a statistically significant intervention effect [76,78,88].

Two studies investigated effectiveness of interventions for preschoolers by implementing a multicomponent intervention [75,88]. Intervention and control group participants in the first study [75] targeting preschoolers reduced time spent sedentary compared to baseline at the 12 month follow-up (IG: 631.3 min/day to 623.9 min/day, CG: 631.4 min/day to 628.1 min/day). Moreover, intervention group participants spent significantly less time per day in sedentary activities at the 12 month follow-up compared to controls (*p* = 0.014). In the second study [88], preschoolers in the intervention group did not spend significantly less time per day sedentary compared to controls (β = −20.30, 95% CI = −42.8 to 2.2) at the 12-month follow-up. However, intervention group participants decreased time spent sedentary compared to baseline (378.7 min/day to 364.4 min/day), while control group participants increased time spent sedentary (362.2 min/day to 369.3 min/day).

In a pan-European study [89] implementing a multicomponent intervention, the percentage of time spent sedentary increased from baseline to the two-year follow-up for boys and girls in intervention and control groups (boys: IG: 35.4 to 40.3, CG: 34.9 to 39.6; girls: IG: 36.5 to 44.1, CG: 37.7 to 41.4). The proportion of time spent sedentary increased significantly over two years for girls in the intervention group compared to controls (effect estimate (EE) = 3.94, *p* = 0.004), while for intervention group boys, no significant effect compared to controls was found (EE = 0,09, *p* = 0.945).

Two studies assessed the effectiveness of interventions in primary school children, one implemented a multicomponent intervention [78] and the other one targeted the physical environment [87]. At the one-year follow-up of the first study [78] targeting school children, children in the intervention and control groups increased their sedentary time compared to baseline (IG: 205 ± 91 min/day to 262 ± 115 min/day, CG: 219 ± 87 min/day to 254 ± 99 min/day), with no significant differences between groups (*p* > 0.05). Results of the second study [87] showed that neither group 1 nor group 2 significantly reduced their total sitting time from baseline to follow-up. Nevertheless, pupils in group 1 spent 8.9 min less time sitting per day (95% CI −44.4 to 32.1), while pupils in group 2 spent 12.6 min more (95% CI −26.9 to 52.0) compared to baseline at follow-up 2.

At the 12-week follow-up of a study [76] implementing a multicomponent intervention targeting teenagers, change in time spent sedentary per day did not reveal a significant difference (*p* = 0.881) between the intervention and control group, although teenagers in both groups spent less time sedentary per day compared to baseline (IG: −0,24 h/day, SE: = 0.25, *p* = 0.346; CG: −0.18 h/day, SE = 0.31, *p* = 0.555).

### 3.5. Meta-Analysis

**Figure 3:**
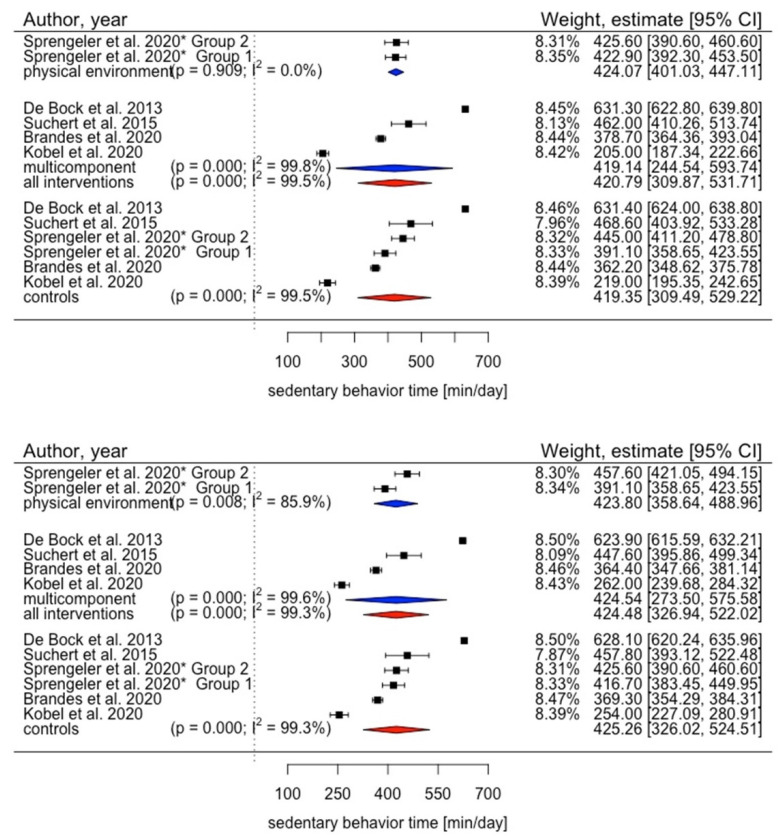
Results of meta-analysis in children [75,76,78,87,88]. Top: sedentary time at baseline. Bottom: sedentary time at study end. * Sprengeler et al., 2020 implemented a cross-over design, therefore each study group served as intervention and control group once. Blue depicts summaries for each intervention type; red depicts overall summaries for (any) intervention and control group.

Meta-analysis of eligible primary studies in children aged 3 to 17 years revealed that pooled sedentary time in intervention groups increased from 420.79 min/day (95% CI 309.87 to 531.71) at baseline to 424.48 min/day (95% CI 326.94 to 522.02) at the end of the study (Figure 3). For control groups, pooled sedentary time increased from 419.35 min/day (95% CI 309.49 to 529.22) to 425.26 min/day (95% CI 326.02 to 524.51) (Figure 3). There was high heterogeneity among the studies (I^2^ (baseline) = 99.5%, I^2^ (study end) = 99.3%). Subgroup analyses revealed pooled sedentary time reduction by physical environment intervention from 424.07 min/day (95% CI 401.03 to 447.11) to 423.80 min/day (95% CI 358.64 to 488.96) in contrast to a sitting time increase following multicomponent intervention from 419.14 min/day (95% CI 244.54 to 593.74) to 424.54 min/day (95% CI 273.50 to 575.58) (Figure 3).

## 4. Discussion

The present systematic review summarized the effectiveness of interventions targeting sedentary time in different target groups and different settings in Germany. Reducing time spent sedentary through effective interventions is important because it may have positive effects on health-related outcomes as current research suggests that replacing sedentary time with more active behaviors may have positive effects on body anthropometry and cardiometabolic risk markers in general [92,93].

Results showed inconsistent evidence regarding change in sedentary time. Our meta-analysis of primary studies targeting children showed that pooled sedentary time increased from baseline to study end in both groups.

Previous reviews concluded that to successfully achieve a reduction in sedentary time, interventions should focus solely on sedentary behavior [22,94]. Moreover, findings indicate that interventions targeting the physical environment are very successful in reducing time spent sedentary, especially at the workplace and at school [22,94,95].

Only one [87] of the included studies implemented a physical environment intervention focusing solely on sedentary behavior. However, that intervention did not lead to a significant reduction in total sitting time per day [87], which may be due to the relatively short intervention duration of two weeks per group and to the cross-over design of the study.

The included studies aiming to decrease sedentary time in children mainly implemented multicomponent interventions, with mixed results. The performed subgroup analysis revealed that time spent sedentary increased from baseline to the study end by multicomponent interventions while it decreased slightly by physical environment interventions. This is consistent with findings showing that for preschoolers and school children, physical and social environment interventions are the most effective [22]. However, those results conflict with findings of another review [95] indicating that in children, interventions using a mixed or behavioral approach are superior to environmental interventions, at least in the short term. However, authors of the latter review [95] differentiated only between behavioral, environmental and mixed approaches and did not consider social environment interventions individually, which limits the comparability of results.

For children, targeting the social environment, particularly the family, appears to contribute decisively to an intervention’s success [96,97]. Active involvement of parents seems to be superior to passive involvement, such as mailing newsletters [97,98]. The only included study that was able to achieve a significant decrease in sedentary time involved a participatory intervention where parents were able to contribute their own project ideas as part of the intervention [75]. As interventions implemented in younger children are associated with greater improvements in sedentary time than interventions in older children [96,97] and because younger children are more strongly influenced by their parents than older children [99], we hypothesize that social environment interventions involving the family are especially successful in younger children.

In two included studies, the intervention led to an increase in sedentary time [78,89]. In both cases, parents were involved mainly passively and the intervention did not focus solely on sedentary behavior. In one study [89], the increase in sedentary time among girls even reached statistical significance. Additionally, the intervention targeted a broad age range, covering toddlers to school children, and it was implemented in eight European countries. Possibly, the intervention did not succeed in sufficiently adapting the intervention to the different age groups and countries.

These findings are reflected by our meta-analysis in children, as pooled sedentary time increased in children in the intervention and control groups. Moreover, the included cross-over study [87] might have distorted the results. Due to the cross-over design, we had to include both study groups as intervention and control group. However, children in the different groups were in the same classroom during the intervention, which may have prompted children in group 2 to reduce their time spent sedentary while serving as control group and to increase their time spent sedentary during the following intervention phase. Moreover, it is conceivable that behaviors build in the intervention phase were maintained in the control phase.

The nine included studies targeting adults used a personal behavior approach, with the exception of one study [85] that used a multicomponent intervention that also included a physical environment component in the form of sit–stand desks. This resulted in a significant decrease in time spent sedentary at work, whereas other studies obtained mixed, statistically non-significant results. These findings suggest that physical environment and multicomponent interventions are more effective than personal behavior interventions to achieve a reduction in workplace or total sitting time for healthy adults. This is in line with the results of a recent umbrella review showing that physical environment and multicomponent interventions are effective in reducing adult sedentary time in the majority of cases [22].

No significant decrease [73,74] or, rather, a statistically non-significant increase, in sedentary time [86] was shown in the three personal behavior interventions targeting people with chronic diseases. This finding conflicts with the results of an umbrella review suggesting that personal behavior interventions effectively reduce sitting time in people with chronic diseases [22]. As only limited data focusing on people with chronic diseases are available, we cannot determine whether these differing findings occurred by chance or are related to conditions specific to German society.

The included study of older adults showed that a personal behavior intervention did not result in a significant decrease in sedentary time [71]. One possible explanation is the lack of an intervention focus on sedentary behavior, which is important in studies targeting older adults [100].

### 4.1. Recommendations to Effectively Reduce Sitting Time in Germany

As various health related outcomes are associated with sedentary behavior and therefore are likely to be influenced by interventions targeting sedentary behavior, one goal of the current review was to provide recommendations on how to effectively reduce sitting time in German society. Due to a limited number of available studies in Germany, our recommendations need to be interpreted with caution.

Interventions targeting the physical and social environment are the most effective intervention types for children [22]. Therefore, for this age group we recommend interventions taking this approach. Regarding physical environment interventions, we propose creating a movement-friendly environment, e.g., through the provision of height adjustable desks in schools [101,102]. When implementing social environment interventions, a focus should be placed on active involvement of the family [97,98]. Furthermore, we suggest intervening at an early age since interventions in younger children are associated with greater improvements in sedentary time [96,97] and sedentary behavior habits seem to track from early childhood to adolescence [103,104,105].

In Germany, adults spend 7.5 h per day sitting, with adults over the age of 65 years spending the least amount of time sitting at 6 h per day. Adults spend 2 h sitting while watching TV, 1.5 h during leisure time and 1.5 h at work. However, 14% of adults spend 6 h per day or more sitting at work and 15% spend between 4 and 6 h per day sitting at work. Overall, two thirds of daily sitting time are accumulated in these settings [19]. To achieve a significant reduction in sitting time at work, physical environment interventions, such as height-adjustable desks, have proven suitable. Appropriate interventions for adults to reduce sedentary time during leisure time target personal behavior [22]. We are unable to recommend specific interventions to reduce sedentary time accumulated while watching TV as evidence in this area is limited and inconclusive [106,107,108].

For older adults, we recommend multicomponent and personal behavior interventions to effectively reduce total sitting time.

Personal behavior interventions targeting people with chronic diseases did not result in a significant reduction in sedentary time, in contrast to international findings [22] where personal behavior interventions effectively reduced sedentary time. Due to a lack of alternative options, we recommend personal behavior interventions but further research is needed.

For overweight or obese people, we recommend implementing personal behavior interventions because they significantly reduce sitting time in this target group [22].

In sum, recommended measures should represent a combination of behavioral and structural prevention approaches. Using structural prevention approaches such as the provision of height-adjustable desks in schools or workplaces improves the effectiveness of individual health-promoting behavior targeted at reducing sedentary time.

### 4.2. Strengths and Limitations

The present review and meta-analysis have various strengths. First, we conducted our review and meta-analysis in accordance with the PRISMA 2020 statement. Moreover, we conducted a comprehensive literature search, including only controlled intervention studies, focusing on sitting time as a sedentary behavior domain and excluding outcomes such as screen time. Additionally, we included data from all settings and diverse target groups, including people with chronic diseases, overweight or obesity. To our knowledge, this is the first systematic review that compares the effectiveness of interventions aiming to reduce sedentary time in Germany.

However, there are some limitations which must be taken into account when interpreting the results. We could not conduct a meta-analysis in adults due to the large heterogeneity in target groups and systematic differences between included studies and the heterogeneity in the meta-analysis in children was high. For that reason and because of the small number of studies implemented in Germany, we were not able to derive recommendations solely from the findings of studies implemented in Germany and needed to consult international evidence. Moreover, the robustness of our findings is limited by the quality of the included studies. The way sedentary time was assessed in the included studies differed. As quantitative and qualitative assessment of sedentary time but also different applied instruments potentially influence obtained sedentary time, bias may occur. Furthermore, few studies report time spent sedentary in specific domains such as at school. It would be important to measure sedentary time during school hours in a standardized way to be able to obtain accurate results. Moreover, risk of bias assessment revealed at least some concerns for all of the evaluated studies. Risk of bias due to missing outcome data was not judged to be low in any of the evaluated studies which may lead to bias in the effect estimates and requires a careful interpretation of results. Furthermore, we included studies with small sample sizes and most studies did not include long-term follow-up.

### 4.3. Implications for Future Research

Additional large-scale and high-quality studies need to be conducted in Germany to derive recommendations for sitting time reduction. Interventions should focus on sedentary behavior itself and not on physical activity or both. Moreover, there is a need for more research in a number of settings and several target groups such as teenagers, preschoolers and people with overweight or obesity. Studies of adults need to specifically address workplace and leisure time settings, and TV viewing in particular, because the majority of time spent sitting is accumulated in those domains. Studies of children and adolescents should focus on physical and social environment interventions. In addition, studies should target preschoolers since early interventions appear promising. Additionally, research is needed to determine whether personal behavior interventions represent a promising approach for people with chronic diseases.

## 5. Conclusions

The present systematic review and meta-analysis summarize the effectiveness of interventions targeting sedentary time in different target groups and different settings in Germany. We found inconsistent evidence with respect to change in sedentary time. We recommend conducting more studies in Germany to create robust evidence. Despite limited data in Germany, we recommend physical and social environment interventions for children. In the workplace setting, we recommend physical environment interventions such as height-adjustable desks.

## Data Availability

Not applicable.

## Supplementary Materials

The following supporting information can be downloaded at: https://www.mdpi.com/article/10.3390/ijerph191610178/s1, Figure S1: PRISMA Checklist, Figure S2: PRISMA Abstract Checklist, Table S1: Full search strategy for PubMed, Table S2: Full search strategy for Web of Science, Table S3: Full search strategy for DRKS, Table S4: List of 35 full-text articles assessed for eligibility that were excluded from the review with reason for exclusion, Table S5: Risk of bias assessment.
