# Supplementary material for: The Effectiveness of Interventions to Reduce Sedentary Time in Different Target Groups and Settings in Germany: Systematic Review, Meta-Analysis and Recommendations on Interventions
Source: Int J Environ Res Public Health. 2022 Aug 17;19(16):10178. doi: 10.3390/ijerph191610178 (PMC9408392; doi:10.3390/ijerph191610178)
Supplement: Supplementary file 1 [file ijerph-19-10178-s001.zip › ijerph-1831919-supplementary.pdf]

The Effectiveness of Interventions to Reduce Sedentary Time in Different Target Groups and Settings in Germany: Systematic Review, Meta-Analysis and Recommendations on Interventions

Nida Mugler *, Hansjörg Baurecht, Kevin Lam, Michael Leitzmann and Carmen Jochem

Department of Epidemiology and Preventive Medicine, University of Regensburg, 93053 Regensburg, Germany
* Correspondence: nida.mugler@stud.uni-regensburg.de; Tel.: +49-941-944-5201

Supplementary Figure S1 PRISMA Checklist, "The effectiveness of interventions to reduce sedentary time in different target groups and settings in Germany: systematic review, meta-analysis and recommendations on interventions"

Section and Topic	Item #	Checklist item	Location where item is reported
**TITLE**			
Title	1	Identify the report as a systematic review.	1
**ABSTRACT**			
Abstract	2	See the PRISMA 2020 for Abstracts checklist.	Supplementary Figure 2
**INTRODUCTION**			
Rationale	3	Describe the rationale for the review in the context of existing knowledge.	1, 2
Objectives	4	Provide an explicit statement of the objective(s) or question(s) the review addresses.	2
**METHODS**			
Eligibility criteria	5	Specify the inclusion and exclusion criteria for the review and how studies were grouped for the syntheses.	2, 3
Information sources	6	Specify all databases, registers, websites, organisations, reference lists and other sources searched or consulted to identify studies. Specify the date when each source was last searched or consulted.	3
Search strategy	7	Present the full search strategies for all databases, registers and websites, including any filters and limits used.	Supplementary Tables 1-3
Selection process	8	Specify the methods used to decide whether a study met the inclusion criteria of the review, including how many reviewers screened each record and each report retrieved, whether they worked independently, and if applicable, details of automation tools used in the process.	3
Data collection process	9	Specify the methods used to collect data from reports, including how many reviewers collected data from each report, whether they worked independently, any processes for obtaining or confirming data from study investigators, and if applicable, details of automation tools used in the process.	3
Data items	10a	List and define all outcomes for which data were sought. Specify whether all results that were compatible with each outcome domain in each study were sought (e.g. for all measures, time points, analyses), and if not, the methods used to decide which results to collect.	3
	10b	List and define all other variables for which data were sought (e.g. participant and intervention characteristics, funding sources). Describe any assumptions made about any missing or unclear information.	3
Study risk of bias assessment	11	Specify the methods used to assess risk of bias in the included studies, including details of the tool(s) used, how many reviewers assessed each study and whether they worked independently, and if applicable, details of automation tools used in the process.	4
Effect measures	12	Specify for each outcome the effect measure(s) (e.g. risk ratio, mean difference) used in the synthesis or presentation of results.	4
Synthesis methods	13a	Describe the processes used to decide which studies were eligible for each synthesis (e.g. tabulating the study intervention characteristics and comparing against the planned groups for each synthesis (item #5)).	3
	13b	Describe any methods required to prepare the data for presentation or synthesis, such as handling of missing summary statistics, or data conversions.	4
	13c	Describe any methods used to tabulate or visually display results of individual studies and syntheses.	3, 4
	13d	Describe any methods used to synthesize results and provide a rationale for the choice(s). If meta-analysis was performed, describe the model(s), method(s) to identify the presence and extent of statistical heterogeneity, and software package(s) used.	3, 4
	13e	Describe any methods used to explore possible causes of heterogeneity among study results (e.g. subgroup analysis, meta-regression).	4
	13f	Describe any sensitivity analyses conducted to assess robustness of the synthesized results.	-

Section and Topic	Item #	Checklist item	Location where item is reported
Reporting bias assessment	14	Describe any methods used to assess risk of bias due to missing results in a synthesis (arising from reporting biases).	-
Certainty assessment	15	Describe any methods used to assess certainty (or confidence) in the body of evidence for an outcome.	-
**RESULTS**			
Study selection	16a	Describe the results of the search and selection process, from the number of records identified in the search to the number of studies included in the review, ideally using a flow diagram.	4, Figure 1
	16b	Cite studies that might appear to meet the inclusion criteria, but which were excluded, and explain why they were excluded.	4, Supplementary Table 4
Study characteristics	17	Cite each included study and present its characteristics.	5-10
Risk of bias in studies	18	Present assessments of risk of bias for each included study.	5, Supplementary Table 5
Results of individual studies	19	For all outcomes, present, for each study: (a) summary statistics for each group (where appropriate) and (b) an effect estimate and its precision (e.g. confidence/credible interval), ideally using structured tables or plots.	10-12
Results of syntheses	20a	For each synthesis, briefly summarise the characteristics and risk of bias among contributing studies.	12,Supplementary Table 5
	20b	Present results of all statistical syntheses conducted. If meta-analysis was done, present for each the summary estimate and its precision (e.g. confidence/credible interval) and measures of statistical heterogeneity. If comparing groups, describe the direction of the effect.	12, 13
	20c	Present results of all investigations of possible causes of heterogeneity among study results.	-
	20d	Present results of all sensitivity analyses conducted to assess the robustness of the synthesized results.	-
Reporting biases	21	Present assessments of risk of bias due to missing results (arising from reporting biases) for each synthesis assessed.	-
Certainty of evidence	22	Present assessments of certainty (or confidence) in the body of evidence for each outcome assessed.	-
**DISCUSSION**			
Discussion	23a	Provide a general interpretation of the results in the context of other evidence.	13-15
	23b	Discuss any limitations of the evidence included in the review.	16
	23c	Discuss any limitations of the review processes used.	16
	23d	Discuss implications of the results for practice, policy, and future research.	15, 16
**OTHER INFORMATION**			
Registration and protocol	24a	Provide registration information for the review, including register name and registration number, or state that the review was not registered.	-
	24b	Indicate where the review protocol can be accessed, or state that a protocol was not prepared.	-
	24c	Describe and explain any amendments to information provided at registration or in the protocol.	-
Support	25	Describe sources of financial or non-financial support for the review, and the role of the funders or sponsors in the review.	17
Competing interests	26	Declare any competing interests of review authors.	17
Availability of data, code and other materials	27	Report which of the following are publicly available and where they can be found: template data collection forms; data extracted from included studies; data used for all analyses; analytic code; any other materials used in the review.	4

From: Page MJ, McKenzie JE, Bossuyt PM, Boutron I, Hoffmann TC, Mulrow CD, et al. The PRISMA 2020 statement: an updated guideline for reporting systematic reviews. BMJ 2021;372:n71. doi: 10.1136/bmj.n71 For more information, visit: http://www.prisma-statement.org/

Supplementary Figure S2 PRISMA Abstract Checklist, "The effectiveness of interventions to reduce sedentary time in different target groups and settings in Germany: systematic review, meta-analysis and recommendations on interventions"

Section and Topic	Item #	Checklist item	Reported (Yes/No)
**TITLE**			
Title	1	Identify the report as a systematic review.	Yes
**BACKGROUND**			
Objectives	2	Provide an explicit statement of the main objective(s) or question(s) the review addresses.	Yes
**METHODS**			
Eligibility criteria	3	Specify the inclusion and exclusion criteria for the review.	Yes
Information sources	4	Specify the information sources (e.g. databases, registers) used to identify studies and the date when each was last searched.	Yes
Risk of bias	5	Specify the methods used to assess risk of bias in the included studies.	n/a
Synthesis of results	6	Specify the methods used to present and synthesise results.	Yes
**RESULTS**			
Included studies	7	Give the total number of included studies and participants and summarise relevant characteristics of studies.	Yes
Synthesis of results	8	Present results for main outcomes, preferably indicating the number of included studies and participants for each. If meta-analysis was done, report the summary estimate and confidence/credible interval. If comparing groups, indicate the direction of the effect (i.e. which group is favoured).	Yes
**DISCUSSION**			
Limitations of evidence	9	Provide a brief summary of the limitations of the evidence included in the review (e.g. study risk of bias, inconsistency and imprecision).	Yes
Interpretation	10	Provide a general interpretation of the results and important implications.	Yes
**OTHER**			
Funding	11	Specify the primary source of funding for the review.	n/a
Registration	12	Provide the register name and registration number.	n/a

From: Page MJ, McKenzie JE, Bossuyt PM, Boutron I, Hoffmann TC, Mulrow CD, et al. The PRISMA 2020 statement: an updated guideline for reporting systematic reviews. BMJ 2021;372:n71. doi: 10.1136/bmj.n71 For more information, visit: http://www.prisma-statement.org/

Supplementary Table S1 Full search strategy for PubMed

Set	Search terms
Sedentary behavior and related interventions	(sedentary[tiab] OR (sit-stand desk[tiab]) OR (active workstation[tiab]))
Include controlled intervention studies only	AND (intervention[tiab] OR control[tiab] OR trial[tiab] OR randomized[tiab])
Include studies implemented in Germany only	AND (german* OR deutsch*)
Limit: exclude pharmacological, animal and cross-sectional studies	NOT (drug OR pharmacolog* OR animal OR (cross-sectional))

Supplementary Table S2 Full search strategy for Web of Science

Set	Search terms
Sedentary behavior and related interventions	(sedentary OR (sit-stand desk) OR (active workstation))
Include controlled intervention studies only	AND (intervention OR control OR trial OR randomized)
Include studies implemented in Germany only	AND (german* OR deutsch*)
Limit: exclude pharmacological, animal and cross-sectional studies	NOT (drug OR pharmacolog* OR animal OR (cross-sectional))
Applied restrictions and filters	Search field: Topic

Supplementary Table S3 Full search strategy for DRKS

Set	Search terms
Sedentary behavior and related interventions	(sedentary behavior) OR (Sitzzeit)
Applied restrictions and filters	Search in German and English trial attributes,<br>Countries of recruitment: Germany

Supplementary Table S4 List of 35 full-text articles assessed for eligibility that were excluded from the review with reason for exclusion

Author, year	Title	Reason for exclusion
Becker et al. 2019 [1]	Children's Cortisol and Cell-Free DNA Trajectories in Relation to Sedentary Behavior and Physical Activity in School: A Pilot Study	Intervention intention is not changing sedentary time
Brehm et al. 2005 [2]	Health promotion by means of health sport--a framework and a controlled intervention study with sedentary adults	Sitting time or sedentary time not reported
De Bourdeaudhuij et al. 2015 [3]	Behavioural effects of a community-oriented setting-based intervention for prevention of childhood obesity in eight European countries. Main results from the IDEFICS study	Only domains of sedentary behavior: screen time, TV viewing, PC/games
Franke et al. 2016 [4]	Telemonitoring of home exercise cycle training in patients with COPD	Sitting time or sedentary time not reported
Gába et al. 2016 [5]	The effect of brisk walking on postural stability, bone mineral density, body weight and composition in women over 50 years with a sedentary occupation: a randomized controlled trial	Sitting time or sedentary time not reported
Hötting et al. 2012 [6]	Long-term effects of physical exercise on verbal learning and memory in middle-aged adults: results of a one-year follow-up study	Sitting time or sedentary time not reported
Kemmler et al. 2020 [7]	Effects of High-Intensity Resistance Training on Osteopenia and Sarcopenia Parameters in Older Men with Osteosarcopenia-One-Year Results of the Randomized Controlled Franconian Osteopenia and Sarcopenia Trial (FrOST)	Sitting time or sedentary time not reported
Knaus et al. 2020 [8]	For better or worse? - The effects of physical education on child development	Only domain of sedentary behavior : media consumption
Kobel et al. 2017 [9]	Effects of a Randomised Controlled School-Based Health Promotion Intervention on Obesity Related Behavioural Outcomes of Children with Migration Background	Only domain of sedentary behavior : screen media use
Konradt et al. 2020 [10]	Beneficial, adverse, and spiraling health-promotion effects: Evidence from a longitudinal randomized controlled trial of working at sit-stand desks	Sitting time or sedentary time not reported
Kraushaar et al. 2014 [11]	Web-Enabled Feedback Control Over Energy Balance Promotes an Increase in Physical Activity and a Reduction of Body Weight and Disease Risk in Overweight Sedentary Adults	Sitting time or sedentary time not reported

Latomme et al. 2017 [12]	Effect and process evaluation of a kindergarten-based, family-involved intervention with a randomized cluster design on sedentary behaviour in 4- to 6- year old European preschool children: The ToyBox-study	Only domains of sedentary behavior : TV/DVD/video viewing, computer/video games use, quiet play
Liersch et al. 2015 [13]	Effektivität und Kosteneffektivität täglichen Schulsports in der Grundschule - das Projekt "fit für pisa"	Only domain of sedentary behavior : TV consumption
Ludwig et al. 2018 [14]	Targeted Athletic Training Improves the Neuromuscular Performance in Terms of Body Posture From Adolescence to Adulthood - Long-Term Study Over 6 Years	Outcome: body posture; report data on sedentary time but sedentary time not defined as outcome of the study
Marschin et al. 2021 [15]	A Short, Multimodal Activity Break Incorporated Into the Learning Context During the Covid-19 Pandemic: Effects of Physical Activity and Positive Expressive Writing on University Students' Mental Health—Results and Recommendations From a Pilot Study	Sedentary time only regarded als indicator for mental health
Miguel-Berges et al. 2020 [16]	Parental perceptions, attitudes and knowledge on European preschool children's total screen time: the ToyBox-study	Only domains of sedentary behavior : TV/video/DVDs viewing and total screen time
Notthoff et al. 2016 [17]	Positive messages enhance older adults' motivation and recognition memory for physical activity programmes	Study setting: Netherlands
Noz et al. 2019 [18]	Sixteen-Week Physical Activity Intervention in Subjects With Increased Cardiometabolic Risk Shifts Innate Immune Function Towards a Less Proinflammatory State	Study setting: Netherlands
Peters et al. 2013 [19]	Internetbasiertes "e-Training" als Bewegungs-intervention zur Gesundheitsförderung: Ergebnisse aus 2 Interventionsstudien	Sitting time or sedentary time not reported
Pressler et al. 2010 [20]	An internet-delivered exercise intervention for workplace health promotion in overweight sedentary employees: a randomized trial	Sitting time or sedentary time not reported
Rank et al. 2014 [21]	Health-related quality of life and physical activity in children and adolescents 2 years after an inpatient weight-loss program	Study design: uncontrolled trial
Ratz et al. 2022 [22]	Distinct physical activity and sedentary behavior trajectories in older adults during participation in a physical activity intervention: a latent class growth analysis	Does not evaluate intervention effectiveness regarding primary outcomes
Reljic et al. 2018 [23]	Effects of low-volume high-intensity interval training in a community setting: a pilot study	Sitting time or sedentary time not reported
Reljic et al. 2020 [24]	Low-volume high-intensity interval training improves cardiometabolic health, work ability and well-being in severely obese individuals: a randomized-controlled trial sub-study	Sitting time or sedentary time not reported

Schwarzfischer et al. 2019 [25]	Physical Activity and Sedentary Behavior From 6 to 11 Years	Study design: cohort study
Schwenk et al. 2019 [26]	The Adapted Lifestyle-Integrated Functional Exercise Program for Preventing Functional Decline in Young Seniors: Development and Initial Evaluation	Study design: uncontrolled pre-post intervention study
Siegrist et al. 2019 [27]	Körperliche Fitness und Gesundheitsverhalten von Kindern in fit4future-Schulen<br>Abschlussbericht nach drei Projektjahren in den Phase-1-Schulen (Zeitraum 2016 bis 2019)	No suitable sedentary behavior domain reported
Smith et al. 2016 [28]	Physical Activity Levels and Domains Assessed by Accelerometry in German Adolescents from GINIplus and LISAplus	Study design: cohort study
Stenner et al. 2020 [29]	Effects of six month personalized endurance training on work ability in middle-aged sedentary women: a secondary analysis of a randomized controlled trial	Sitting time or sedentary time not reported
Streber et al. 2017 [30]	A multicenter controlled study for dementia prevention through physical, cognitive and social activities - GESTALT-kompakt	Sitting time or sedentary time not reported
Szczuka et al. 2021 [31]	Can individual, dyadic, or collaborative planning reduce sedentary behavior? A randomized controlled trial	Study setting: Poland
Vik et al. 2015 [32]	Evaluation of the UP4FUN intervention: a cluster randomized trial to reduce and break up sitting time in European 10-12-year-old children	Sedentary time not separately reported for Germany
Wang et al. 2021 [33]	A Smartphone App to Support Sedentary Behavior Change by Visualizing Personal Mobility Patterns and Action Planning (SedVis): Development and Pilot Study	Given sedentary time includes sleeping time
Wolff et al. 2014 [34]	What do targeting positive views on ageing add to a physical activity intervention in older adults? Results from a randomised controlled trial	Sitting time or sedentary time not reported
Zusman et al. 2019 [35]	Older Adults' Sedentary Behavior and Physical Activity After Hip Fracture: Results From an Outpatient Rehabilitation Randomized Controlled Trial	Study setting: Canada

Some studies were excluded for multiple reasons, to preserve clarity we only listed one reason in these cases.

Supplementary Table S5 Risk of bias assessment using RoB 2

Author, year	Study design	Experimental	Comparator	Outcome	Randomization process		Deviations intended intervention	Missing outcome data	Measurement of outcome	Selection of the reported results	Overall risk of bias
Voigt et al. 2018 [36]	RCT	Tailored letters	Assessment only	Sedentary time	Some concerns		Low risk	High risk	High risk	Some concerns	High risk
Livingstone et al. 2020 [37]	RCT	Personalized advice	General advice	Sedentary time	Some concerns		Low risk	Some concerns	Low risk	Some concerns	Some concerns
Ellegast et al. 2012 [38]	RCT	Multicomponent intervention	No intervention	Sitting time	Some concerns		High risk	High risk	High risk	Some concerns	High risk
Muellmann et al. 2019 [39]	RCT	IG1: group meetings, material, website<br>IG2: IG1 + activity tracker	Delayed intervention	Sedentary time	Some concerns		Low risk	Some concerns	Low risk	Some concerns	Some concerns
Kleinke et al. 2021 [40]	RCT	Personalized feedback, official recommendations	Delayed feedback	Sedentary time	Low risk		Low risk	High risk	Some concerns	Low risk	High risk
Salchow et al. 2021 [41]	RCT	Tailored PA counselling	Usual care	Sitting	Low risk		High risk	High risk	High risk	Some concerns	High risk
Geidl et al. 2021 [42]	RCT	Stationary rehabilitation, pedometer, PA lessons	Stationary rehabilitation, repetition PA information	Sedentary time	Low risk		Low risk	Some concerns	Low risk	Low risk	Some concerns
Wagner et al. 2019 [43]	RCT	Stationary rehabilitation + personal behavior intervention	Stationary rehabilitation	Sedentary time	Some concerns		High risk	High risk	Low risk	Some concerns	High risk
De Bock et al. 2013 [44]	Cluster-RCT	Non-participatory PA program + participatory intervention	Non-participatory PA program	Sedentary time	Low risk	* Low risk	Low risk	Some concerns	Low risk	Some concerns	Some concerns
Kobel et al. 2020 [45]	Cluster-RCT	Multicomponent intervention	No intervention	Sedentary time	Some concerns	* Low risk	High risk	High risk	Low risk	Some concerns	High risk
Suchert et al. 2015 [46]	Cluster-RCT	Multicomponent intervention	Regular education	Sedentary time	Some concerns	* Some concerns	Low risk	High risk	High risk	Low risk	High risk
Sprengeler et al. 2020 [47]	Cross-over RCT	height-adjustable standing desks	traditional working desks	Sitting time	Some concerns	**S** High risk	High risk	Some concerns	Low risk	Some concerns	High risk
Pischke et al. 2022 [48]	Cross-over RCT	WEB: online materials, group sessions<br>WEB+: WEB + activity tracker	PRINT: printed materials, group sessios	Sedentary time	Some concerns	**S** Some concerns	Low risk	High risk	Low risk	Some concerns	High risk

IG = Intervention group, PA = physical activity, RCT = Randomized controlled trial * for Cluster-RCT: risk of bias arising from timing of identification or recruitment, **S** = Domain S for crossover RCT: risk of bias arising from period and carryover effects

References

1. Becker, C.; Schmidt, S.; Neuberger, E.W.I.; Kirsch, P.; Simon, P.; Dettweiler, U. Children's Cortisol and Cell-Free DNA Trajectories in Relation to Sedentary Behavior and Physical Activity in School: A Pilot Study. *Front. Public Health* **2019**, *7*, 26, doi:10.3389/fpubh.2019.00026.
2. Brehm, W.; Wagner, P.; Sygusch, R.; Schönung, A.; Hahn, U. Health promotion by means of health sport--a framework and a controlled intervention study with sedentary adults. *Scand. J. Med. Sci. Sports* **2005**, *15*, 13–20, doi:10.1111/j.1600-0838.2003.00369.x.
3. Bourdeaudhuij, I. de; Verbestel, V.; Henauw, S. de; Maes, L.; Huybrechts, I.; Mårild, S.; Eiben, G.; Moreno, L.A.; Barba, G.; Kovács, É.; et al. Behavioural effects of a community-oriented setting-based intervention for prevention of childhood obesity in eight European countries. Main results from the IDEFICS study. *Obes. Rev.* **2015**, *16 Suppl 2*, 30–40, doi:10.1111/obr.12347.
4. Franke, K.-J.; Domanski, U.; Schroeder, M.; Jansen, V.; Artmann, F.; Weber, U.; Ettler, R.; Nilius, G. Telemonitoring of home exercise cycle training in patients with COPD. *Int. J. Chron. Obstruct. Pulmon. Dis.* **2016**, *11*, 2821–2829, doi:10.2147/COPD.S114181.
5. Gába, A.; Cuberek, R.; Svoboda, Z.; Chmelík, F.; Pelclová, J.; Lehnert, M.; Frömel, K. The effect of brisk walking on postural stability, bone mineral density, body weight and composition in women over 50 years with a sedentary occupation: a randomized controlled trial. *BMC Womens. Health* **2016**, *16*, 63, doi:10.1186/s12905-016-0343-1.
6. Hötting, K.; Schauenburg, G.; Röder, B. Long-term effects of physical exercise on verbal learning and memory in middle-aged adults: results of a one-year follow-up study. *Brain Sci.* **2012**, *2*, 332–346, doi:10.3390/brainsci2030332.
7. Kemmler, W.; Kohl, M.; Fröhlich, M.; Jakob, F.; Engelke, K.; Stengel, S. von; Schoene, D. Effects of High-Intensity Resistance Training on Osteopenia and Sarcopenia Parameters in Older Men with Osteosarcopenia-One-Year Results of the Randomized Controlled Franconian Osteopenia and Sarcopenia Trial (FrOST). *J. Bone Miner. Res.* **2020**, *35*, 1634–1644, doi:10.1002/jbmr.4027.
8. Knaus, M.C.; Lechner, M.; Reimers, A.K. For better or worse? - The effects of physical education on child development. *Labour Economics* **2020**, *67*, doi:10.1016/j.labeco.2020.101904.
9. Kobel, S.; Lämmle, C.; Wartha, O.; Kesztyüs, D.; Wirt, T.; Steinacker, J.M. Effects of a Randomised Controlled School-Based Health Promotion Intervention on Obesity Related Behavioural Outcomes of Children with Migration Background. *J. Immigr. Minor. Health* **2017**, *19*, 254–262, doi:10.1007/s10903-016-0460-9.
10. Konradt, U.; Heblich, F.; Krys, S.; Garbers, Y.; Otte, K.-P. Beneficial, adverse, and spiraling health-promotion effects: Evidence from a longitudinal randomized controlled trial of working at sit-stand desks. *J. Occup. Health Psychol.* **2020**, *25*, 68–81, doi:10.1037/ocp0000161.
11. Kraushaar, L.E.; Krämer, A. Web-enabled feedback control over energy balance promotes an increase in physical activity and a reduction of body weight and disease risk in overweight sedentary adults. *Prev. Sci.* **2014**, *15*, 579–587, doi:10.1007/s11121-013-0398-2.
12. Latomme, J.; Cardon, G.; Bourdeaudhuij, I. de; Iotova, V.; Koletzko, B.; Socha, P.; Moreno, L.; Androutsos, O.; Manios, Y.; Craemer, M. de. Effect and process evaluation of a kindergarten-based, family-involved intervention with a randomized cluster design on sedentary behaviour in 4- to 6- year old European preschool children: The ToyBox-study. *PLoS One* **2017**, *12*, e0172730, doi:10.1371/journal.pone.0172730.
13. Liersch, S.; Henze, V.; Sterdt, E.; Sayed, M.; Röbl, M.; Schnitzerling, J.; Suermann, T.; Mayr, E.; Krauth, C.; Walter, U. Effectiveness and Cost-Effectiveness of Daily School Sport in the Primary School--Project: "fit for pisa". *Gesundheitswesen* **2015**, *77 Suppl 1*, S72-3, doi:10.1055/s-0032-1331251.
14. Ludwig, O.; Kelm, J.; Hammes, A.; Schmitt, E.; Fröhlich, M. Targeted Athletic Training Improves the Neuromuscular Performance in Terms of Body Posture From Adolescence to Adulthood - Long-Term Study Over 6 Years. *Front. Physiol.* **2018**, *9*, 1620, doi:10.3389/fphys.2018.01620.

15. Marschin, V.; Herbert, C. A Short, Multimodal Activity Break Incorporated Into the Learning Context During the Covid-19 Pandemic: Effects of Physical Activity and Positive Expressive Writing on University Students' Mental Health-Results and Recommendations From a Pilot Study. *Front. Psychol.* **2021**, *12*, 645492, doi:10.3389/fpsyg.2021.645492.
16. Miguel-Berges, M.L.; Santaliestra-Pasias, A.M.; Mouratidou, T.; Flores-Barrantes, P.; Androutsos, O.; Craemer, M. de; Galcheva, S.; Koletzko, B.; Kulaga, Z.; Manios, Y.; et al. Parental perceptions, attitudes and knowledge on European preschool children's total screen time: the ToyBox-study. *Eur. J. Public Health* **2020**, *30*, 105–111, doi:10.1093/eurpub/ckz151.
17. Notthoff, N.; Klomp, P.; Doerwald, F.; Scheibe, S. Positive messages enhance older adults' motivation and recognition memory for physical activity programmes. *Eur. J. Ageing* **2016**, *13*, 251–257, doi:10.1007/s10433-016-0368-1.
18. Noz, M.P.; Hartman, Y.A.W.; Hopman, M.T.E.; Willems, Peter H G M; Tack, C.J.; Joosten, L.A.B.; Netea, M.G.; Thijssen, D.H.J.; Riksen, N.P. Sixteen-Week Physical Activity Intervention in Subjects With Increased Cardiometabolic Risk Shifts Innate Immune Function Towards a Less Proinflammatory State. *J. Am. Heart Assoc.* **2019**, *8*, e013764, doi:10.1161/JAHA.119.013764.
19. Peters, S.; Hentschke, C.; Pfeifer, K. Internet-based "e-training" as exercise intervention for health promotion: results from 2 intervention studies. *Rehabilitation (Stuttg)* **2013**, *52*, 173–181, doi:10.1055/s-0033-1343490.
20. Pressler, A.; Knebel, U.; Esch, S.; Kölbl, D.; Esefeld, K.; Scherr, J.; Haller, B.; Schmidt-Trucksäss, A.; Krcmar, H.; Halle, M.; et al. An internet-delivered exercise intervention for workplace health promotion in overweight sedentary employees: a randomized trial. *Prev. Med.* **2010**, *51*, 234–239, doi:10.1016/j.ypmed.2010.07.008.
21. Rank, M.; Wilks, D.C.; Foley, L.; Jiang, Y.; Langhof, H.; Siegrist, M.; Halle, M. Health-Related Quality of Life and Physical Activity in Children and Adolescents 2 Years after an Inpatient Weight-Loss Program. *Journal of Pediatrics* **2014**, *165*, 732-U397, doi:10.1016/j.jpeds.2014.05.045.
22. Ratz, T.; Pischke, C.R.; Voelcker-Rehage, C.; Lippke, S. Distinct physical activity and sedentary behavior trajectories in older adults during participation in a physical activity intervention: a latent class growth analysis. *Eur. Rev. Aging Phys. Act.* **2022**, *19*, 1, doi:10.1186/s11556-021-00281-x.
23. Reljic, D.; Wittmann, F.; Fischer, J.E. Effects of low-volume high-intensity interval training in a community setting: a pilot study. *Eur. J. Appl. Physiol.* **2018**, *118*, 1153–1167, doi:10.1007/s00421-018-3845-8.
24. Reljic, D.; Frenk, F.; Herrmann, H.J.; Neurath, M.F.; Zopf, Y. Low-volume high-intensity interval training improves cardiometabolic health, work ability and well-being in severely obese individuals: a randomized-controlled trial sub-study. *J. Transl. Med.* **2020**, *18*, 419, doi:10.1186/s12967-020-02592-6.
25. Schwarzfischer, P.; Gruszfeld, D.; Stolarczyk, A.; Ferre, N.; Escribano, J.; Rousseaux, D.; Moretti, M.; Mariani, B.; Verduci, E.; Koletzko, B.; et al. Physical Activity and Sedentary Behavior From 6 to 11 Years. *Pediatrics* **2019**, *143*, doi:10.1542/peds.2018-0994.
26. Schwenk, M.; Bergquist, R.; Boulton, E.; van Ancum, J.M.; Nerz, C.; Weber, M.; Barz, C.; Jonkman, N.H.; Taraldsen, K.; Helbostad, J.L.; et al. The Adapted Lifestyle-Integrated Functional Exercise Program for Preventing Functional Decline in Young Seniors: Development and Initial Evaluation. *Gerontology* **2019**, *65*, 362–374, doi:10.1159/000499962.
27. Siegrist, M.; Schönfeld, J. Körperliche Fitness und Gesundheitsverhalten von Kindern in fit4future- Schulen: Abschlussbericht nach drei Projektjahren in den Phase-1-Schulen (Zeitraum 2016 bis 2019). Available online: https://www.dak.de/dak/download/bericht-2237170.pdf (accessed on 10 November 2021).
28. Smith, M.P.; Berdel, D.; Nowak, D.; Heinrich, J.; Schulz, H. Physical Activity Levels and Domains Assessed by Accelerometry in German Adolescents from GINIplus and LISAplus. *PLoS One* **2016**, *11*, doi:10.1371/journal.pone.0152217.
29. Stenner, H.T.; Eigendorf, J.; Kerling, A.; Kueck, M.; Hanke, A.A.; Boyen, J.; Nelius, A.-K.; Melk, A.; Boethig, D.; Bara, C.; et al. Effects of six month personalized endurance training on work ability in middle-aged sedentary women: a secondary analysis of a randomized controlled trial. *J. Occup. Med. Toxicol.* **2020**, *15*, 8, doi:10.1186/s12995-020-00261-4.

30. Streber, A.; Abu-Omar, K.; Hentschke, C.; Rütten, A. A multicenter controlled study for dementia prevention through physical, cognitive and social activities - GESTALT-kompakt. *Clin. Interv. Aging* **2017**, *12*, 2109–2121, doi:10.2147/CIA.S141163.
31. Szczuka, Z.; Kulis, E.; Boberska, M.; Banik, A.; Kruk, M.; Keller, J.; Knoll, N.; Scholz, U.; Abraham, C.; Luszczynska, A. Can individual, dyadic, or collaborative planning reduce sedentary behavior? A randomized controlled trial. *Social Science & Medicine* **2021**, *287*, 114336, doi:10.1016/j.socscimed.2021.114336.
32. Vik, F.N.; Lien, N.; Berntsen, S.; Bourdeaudhuij, I. de; Grillenberger, M.; Manios, Y.; Kovacs, E.; Chinapaw, M.J.M.; Brug, J.; Bere, E. Evaluation of the UP4FUN intervention: a cluster randomized trial to reduce and break up sitting time in European 10-12-year-old children. *PLoS One* **2015**, *10*, e0122612, doi:10.1371/journal.pone.0122612.
33. Wang, Y.; König, L.M.; Reiterer, H. A Smartphone App to Support Sedentary Behavior Change by Visualizing Personal Mobility Patterns and Action Planning (SedVis): Development and Pilot Study. *JMIR Form. Res.* **2021**, *5*, e15369, doi:10.2196/15369.
34. Wolff, J.K.; Warner, L.M.; Ziegelmann, J.P.; Wurm, S. What do targeting positive views on ageing add to a physical activity intervention in older adults? Results from a randomised controlled trial. *Psychol. Health* **2014**, *29*, 915–932, doi:10.1080/08870446.2014.896464.
35. Zusman, E.Z.; Dawes, M.; Fleig, L.; McAllister, M.M.; Cook, W.L.; Guy, P.; Brasher, P.M.A.; McKay, H.A.; Khan, K.M.; Ashe, M.C. Older Adults' Sedentary Behavior and Physical Activity After Hip Fracture: Results From an Outpatient Rehabilitation Randomized Controlled Trial. *J. Geriatr. Phys. Ther.* **2019**, *42*, E32-E38, doi:10.1519/JPT.0000000000000193.
36. Voigt, L.; Baumann, S.; Ullrich, A.; Weymar, F.; John, U.; Ulbricht, S. The effect of mere measurement from a cardiovascular examination program on physical activity and sedentary time in an adult population. *BMC Sports Sci. Med. Rehabil.* **2018**, *10*, 1, doi:10.1186/s13102-018-0090-8.
37. Livingstone, K.M.; Celis-Morales, C.; Navas-Carretero, S.; San-Cristobal, R.; Forster, H.; Woolhead, C.; O'Donovan, C.B.; Moschonis, G.; Manios, Y.; Traczyk, I.; et al. Characteristics of participants who benefit most from personalised nutrition: findings from the pan-European Food4Me randomised controlled trial. *Br. J. Nutr.* **2020**, *123*, 1396–1405, doi:10.1017/S0007114520000653.
38. Ellegast, R.; Weber, B.; Mahlberg, R. Method inventory for assessment of physical activity at VDU workplaces. *Work* **2012**, *41 Suppl 1*, 2355–2359, doi:10.3233/WOR-2012-0464-2355.
39. Muellmann, S.; Buck, C.; Voelcker-Rehage, C.; Bragina, I.; Lippke, S.; Meyer, J.; Peters, M.; Pischke, C.R. Effects of two web-based interventions promoting physical activity among older adults compared to a delayed intervention control group in Northwestern Germany: Results of the PROMOTE community-based intervention trial. *Prev. Med. Rep.* **2019**, *15*, 100958, doi:10.1016/j.pmedr.2019.100958.
40. Kleinke, F.; Ulbricht, S.; Dorr, M.; Penndorf, P.; Hoffmann, W.; van den Berg, N. A low-threshold intervention to increase physical activity and reduce physical inactivity in a group of healthy elderly people in Germany: Results of the randomized controlled MOVING study. *PLoS One* **2021**, *16*, doi:10.1371/journal.pone.0257326.
41. Salchow, J.; Koch, B.; Mann, J.; Grundherr, J. von; Elmers, S.; Dwinger, S.; Escherich, G.; Vettorazzi, E.; Reer, R.; Sinn, M.; et al. Effects of a structured counselling-based intervention to improve physical activity behaviour of adolescents and young adult cancer survivors - the randomized phase II Motivate AYA - MAYA trial. *Clinical Rehabilitation* **2021**, 269215521997974, doi:10.1177/0269215521997974.
42. Geidl, W.; Carl, J.; Schuler, M.; Mino, E.; Lehbert, N.; Wittmann, M.; Pfeifer, K.; Schultz, K. Long-Term Benefits of Adding a Pedometer to Pulmonary Rehabilitation for COPD: The Randomized Controlled STAR Trial. *Int. J. Chron. Obstruct. Pulmon. Dis.* **2021**, *16*, 1977–1988, doi:10.2147/COPD.S304976.
43. Wagner, P.; Gimpel, C.; Müller, K.; Noack, E.; Bieber, S.; König, S. Abschlussbericht zum Vorhaben "Entwicklung und Evaluation einer verhaltensorientierten Bewegungsintervention zur Aufrechterhaltung der körperlichen Aktivität sowie deren Wirkung auf die psychische Gesundheit von Patienten*innen mit berufsbedingten Lungen- und Atemwegserkrankungen in Kooperation mit der BG Klinik für Berufskrankheiten Falkenstein". Available online: https://docplayer.org/185947303-Abschlussbericht-zum-vorhaben.html (accessed on 13 October 2021).
44. Bock, F. de; Genser, B.; Raat, H.; Fischer, J.E.; Renz-Polster, H. A participatory physical activity intervention in preschools: a cluster randomized controlled trial. *Am. J. Prev. Med.* **2013**, *45*, 64–74, doi:10.1016/j.amepre.2013.01.032.

45. Kobel, S.; Dreyhaupt, J.; Wartha, O.; Kettner, S.; Hoffmann, B.; Steinacker, J.M. Intervention Effects of the Health Promotion Programme "Join the Healthy Boat" on Objectively Assessed Sedentary Time in Primary School Children in Germany. *Int. J. Environ. Res. Public Health* **2020**, *17*, doi:10.3390/ijerph17239029.
46. Suchert, V.; Isensee, B.; Sargent, J.; Weisser, B.; Hanewinkel, R. Prospective effects of pedometer use and class competitions on physical activity in youth: A cluster-randomized controlled trial. *Prev. Med.* **2015**, *81*, 399–404, doi:10.1016/j.ypmed.2015.10.002.
47. Sprengeler, O.; Hebestreit, A.; Gohres, H.; Bucksch, J.; Buck, C. Effects of Installing Height-Adjustable Standing Desks on Daily and Domain-Specific Duration of Standing, Sitting, and Stepping in 3rd Grade Primary School Children. *Front. Public Health* **2020**, *8*, 396, doi:10.3389/fpubh.2020.00396.
48. Pischke, C.R.; Voelcker-Rehage, C.; Ratz, T.; Peters, M.; Buck, C.; Meyer, J.; Holdt, K. von; Lippke, S. Web-Based Versus Print-Based Physical Activity Intervention for Community-Dwelling Older Adults: Crossover Randomized Trial. *JMIR Mhealth Uhealth* **2022**, *10*, e32212, doi:10.2196/32212.
